# Assessing the Effect of CeO_2_ Nanoparticles as Corrosion Inhibitor in Hybrid Biobased Waterborne Acrylic Direct to Metal Coating Binders

**DOI:** 10.3390/polym13060848

**Published:** 2021-03-10

**Authors:** Edurne González, Robin Stuhr, Jesús Manuel Vega, Eva García-Lecina, Hans-Jürgen Grande, Jose Ramon Leiza, María Paulis

**Affiliations:** 1 POLYMAT, Applied Chemistry Department, Faculty of Chemistry, University of the Basque Country (UPV/EHU), 20018 Donostia-San Sebastián, Spain; edurne.gonzalezg@ehu.eus (E.G.); robin.stuhr@studium.uni-hamburg.de (R.S.); jrleiza@ehu.eus (J.R.L.); 2 CIDETEC, Basque Research and Technology Alliance (BRTA), Paseo Miramón 196, 20014 Donostia-San Sebastián, Spain; jvega@cidetec.es (J.M.V.); egarcia@cidetec.es (E.G.-L.); hgrande@cidetec.es (H.-J.G.); 3 POLYMAT, Polymers and Advanced Materials: Physics, Chemistry and Technology Department, Faculty of Chemistry, University of the Basque Country (UPV/EHU), 20018 Donostia-San Sebastián, Spain

**Keywords:** waterborne binder, anticorrosion, biobased acrylic binder, CeO_2_/acrylic hybrid, CeO_2_ nanoparticles, EIS

## Abstract

CeO_2_ nanoparticles were incorporated in waterborne binders containing high biobased content (up to 70%) in order to analyze the anticorrosion performance for direct to metal coatings. Biobased binders were synthesized by batch miniemulsion polymerization of 2-octyl acrylate and isobornyl methacrylate monomers using a phosphate polymerizable surfactant (Sipomer PAM200) that lead to the formation of phosphate functionalized latexes. Upon the direct application of such binders on steel, the functionalized polymer particles were able to interact with steel, creating a thin phosphatization layer between the metal and the polymer and avoiding flash rust. The in situ incorporation of the CeO_2_ nanoparticles during the polymerization process led to their homogeneous distribution in the final polymer film, which produced outstanding anticorrosion performance according to the Electrochemical Impedance Spectroscopy measurements. In fact, steel substrates coated with the hybrid polymer film (30–40 µm thick) showed high barrier corrosion resistance after 41 days (~1000 h) of immersion in NaCl water solution and active inhibition capabilities thanks to the presence of the CeO_2_ nanoparticles. This work opens the door to the fabrication of sustainable hybrid anticorrosion waterborne coatings.

## 1. Introduction

Nowadays, mild steel is one of the most important materials in construction, industry and transportation because of its versatility and good mechanical properties. Nevertheless, the main drawback of steel is its susceptibility to deterioration by corrosion, which causes dramatic economic losses (3.4% of the global GDP in 2013 according to NACE [1]). Therefore, the development of a successful protective organic coating is still an important scientific challenge [2].

An efficient anticorrosion coating must offer good barrier properties, in order to avoid the contact of the steel with water and oxygen (i.e., hindering their permeability). Such barrier capabilities are mainly provided by the polymer matrix in organic coatings, where solvent-based polymers are the most popular among the commercial ones. However, due to the more and more demanding environmental regulations on the emission of volatile organic compounds (VOC), the coating market is moving towards the use of waterborne coatings.

Waterborne coatings are based on polymer latexes, and even if they are an excellent environmentally friendly alternative to solvent-based coatings, their main drawback is the inherent higher hydrophilicity of the formed films due to the presence of surfactants and salts (needed for the synthesis of the latex). Films cast from waterborne latexes have shown to present higher permeability to water than the ones cast from solvent-based systems [3,4,5]. This is detrimental to achieve a good anticorrosion protection. Water permeability can be reduced by the use of polymerizable surfactants (also called surfmers). This type of surfactant is chemically bonded to the polymer particle; and therefore, their migration during the film formation is restricted, avoiding the formation of hydrophilic pockets in the film and improving its water resistance [6,7,8]. Chimenti et al. created a steel protective coating based on an acrylic latex (made of a copolymer of methyl methacrylate (MMA) and butyl acrylate (BA)) stabilized by a phosphate functionalized polymerizable surfactant (Sipomer-PAM200) [9]. This coating showed a lower water sensitivity compared to one stabilized with a conventional anionic surfactant; additionally, the phosphate groups of the polymerizable surfactant were able to react with the metal surface, forming an iron phosphate layer at the substrate/coating interface that provided a great corrosion resistance even in harsh conditions. In later works, the barrier properties of the phosphate functionalized acrylic latexes were improved either by introducing crystalline nanodomains [10] or incorporating 30% of perfluorooctyl acrylate (POA) [11]. 

Nevertheless, an important challenge while designing an environmentally friendly waterborne coating is the replacement of oil-based monomers by biobased ones to reduce the overall carbon footprint of the final product, while maintaining or improving its performance. In fact, the market demand of biobased paints and coatings has constantly increased in the last years [12,13]. The use of different types of biobased monomers to produce waterborne coatings has been extensively reviewed in the literature [14,15]. Even if several works have been published on the use of biobased monomers in emulsion and miniemulsion polymerization, few of them have used commercially available monomers, which makes the industrial implementation of the process difficult. To this end, one of the objectives of this work has been to use commercially available high biobased content monomers, namely 2-octyl acrylate (2-OA) and isobornyl methacrylate (IBOMA), in order to produce a biobased acrylic binder. 2-OA is a monomer derived from castor oil that has a biocontent of 73%. IBOMA comes from pine resin and has a biocontent of 71%. Both monomers have been previously used for the fabrication of biobased Pressure Sensitive Adhesives (PSA) [16,17,18,19] and coatings [20], producing polymers with superior hydrophobic character than conventional MMA/BA copolymers.

Apart from the barrier properties, anticorrosion properties of coatings can also be improved by adding corrosion inhibitors. Most chemical inhibitors reduce the rate of corrosion forming a passive adsorption layer on the metal surface [21]. Chromate based inhibitors incorporated in coatings are known to be the most efficient anticorrosive method for a large range of metals and alloys, reducing both the anodic and cathodic reactions that result in corrosion and metal loss [22]. However, hexavalent chromium was banned due to its high toxicity; thus, alternative and non-toxic chemical inhibitors are needed in order to replace these highly efficient chromate-based compounds. In the last decades, new inorganic and organic inhibitors have been investigated. Either anodic or cathodic inhibition mechanism can be found in corrosion inhibitors [23,24]. In the case of inorganic ones, ion-exchange pigments (e.g., cation-exchange [25,26,27] or anion-exchange ones [28,29]) and nanoparticles (e.g., cerium oxide (CeO_2_) [30,31,32,33], silica (SiO_2_) [34] and zinc oxide (ZnO) [35,36]) have shown promising results as corrosion inhibitors. In the case of the cerium compounds, the inhibiting effect of cerium salts has been outlined by various authors [37,38,39,40], and it is under debate if the nanoparticles as such provide inhibiting effect. In any case, it seems that the more homogeneously the nanoparticles are distributed in the polymeric film, the better the anticorrosion performance [36,41,42].

In this work, and for the first time, a high biobased content waterborne anticorrosion binder containing a phosphatizing agent and CeO_2_ nanoparticles as inhibitor have been successfully synthesized and assessed for the production of direct to metal sustainable coatings.

## 2. Materials and Methods

### 2.1. Materials

IBOMA (Evonik, Essen, Germany) and 2-OA (Arkema, Colombes, France) monomers were used as supplied. The thermal initiator azobisisobutyronitrile (AIBN, Sigma-Aldrich, Madrid, Spain) and the polymerizable surfactant Sipomer^®^ PAM200 (Solvay) were used as received. Octadecyl acrylate (Sigma-Aldrich, Madrid, Spain) was used as co-stabilizer during the miniemulsion polymerization. A solution of CeO_2_ nanoparticles (NANO BYK 3812) was kindly supplied by ALTANA (Wesel, Germany). In order to obtain the pure nanoparticles, the solvent was evaporated in an oven at 60 °C for 48 h. The resulting crystals were grinded before their use. Distilled water (MilliQ quality) was used in all reactions. Sodium bicarbonate (Sigma-Aldrich, Madrid, Spain) and ammonium hydroxide solution (25%, Sigma-Aldrich, Madrid, Spain) were used to adjust pH values. Steel substrates (medium carbon steel with 0.5% of C) were purchased from URDURI ACEROS. UniClean 251 (Atotech, Erandio, Spain) was used as a degreasing agent for the steel substrates. HCl 1 M solution (Sigma-Aldrich, Madrid, Spain) was used in the cleaning treatment of the steel substrates. High purity NaCl (Corrosalt, Ascott-Analytical, Tamworth, UK) was used for the preparation of a 3.5 wt% solution for the corrosion test.

### 2.2. Synthesis and Characterization of Latexes

Two different latexes (without and with 1 wbm % of CeO_2_, named Bioacrylic and CeO_2_-Bioacrylic, respectively) were synthesized by batch miniemulsion polymerization. The used recipe is shown in Table 1. For the miniemulsion preparation, the oil phase was prepared by mixing the main monomers (IBOMA/2-OA), the monomeric costabilizer (octadecyl acrylate) and the dried cerium dioxide (CeO_2_) powder. This mixture was stirred magnetically for 5 min. The aqueous phase was obtained by dissolving the Sipomer^®^ PAM200 in water and adjusting the pH to 7 adding ammonium hydroxide dropwise. Both phases were mixed for 5 min under magnetic agitation and then sonified for 20 min using a Branson 450 w. During sonication, the flask was immersed in an ice bath to avoid overheating. The miniemulsion was later charged into a 0.5 L glass jacketed reactor fitted with a reflux condenser, a sampling device, a N_2_ inlet and a stirrer rotating at 150 rpm. The temperature was controlled by an automatic control system (Camile TG, CRW Automation Solutions, Austin, USA). After reaching the desired temperature (70 °C), a shot of AIBN initiator was added. The reaction was carried out for three hours.

Conversion of the final latexes was measured gravimetrically. Dynamic Light Scattering (DLS, Zetasizer Nano ZS, Malvern Instruments, Malvern, UK) was used to measure the z-average diameter of the miniemulsion droplets and final polymer particles. The morphology of the final latex particles and of the cryosectioned films was analyzed by Transmission Electron Microscopy (TEM) in a TECNAI G2 20 TWIN (FEI) operating at an accelerating voltage of 200 kV in a bright field image mode. Polymer particles and films were observed without any staining.

### 2.3. Film Formation and Properties

For water uptake experiments, the films were formed by casting the latexes (around 1.5 g) onto round silicone molds and drying them at 24 ± 2 °C and 50 ± 5% relative humidity during 6 days until a constant weight was achieved. These films were carefully peeled from the silicone mold and immersed in distilled water during fourteen days. Films were withdrawn from the water container at 24 h intervals, they were smoothly dried and quickly weighed.

For the water static contact angle and EIS measurements, 90 µm wet thick films were cast onto steel plates. Steel substrates were degreased with UniClean 251 solution at 70 °C in a shaking bath for 5 min, followed by 1 min pickling in HCl solution (1:1). Then, the waterborne latexes were uniformly applied on the steel substrates using a quadruple film applicator (Khushbooscientific). Films were dried at a relative humidity of 60% and a temperature of 23 °C for 24 h using a humidity chamber (ESPEC SH-641 benchtop type). The contact angle of the film–air interface was measured in a Contact Angle System OCA (Dataphysics, Filderstadt, Germany) equipment, taking an average value from 20 measurements.

A potentiostat (brand BIO-LOGIC, model VMP3, Seyssinet-Pariset, France) was used to evaluate the corrosion behaviour of the systems by electrochemical measurements: open circuit potential (OCP) and EIS. The following three electrodes configuration was used: Ag/AgCl saturated with KCl as reference electrode, platinum mesh as a counter electrode and coated steel specimens as working electrode. OCP and EIS tests were conducted in 3.5 wt% NaCl solution at room temperature at least by triplicate, using an area of 1 cm^2^. Although OCP was monitored continuously with time, it was interrupted to carry out EIS measurements (once per hour). The frequency range was from 100 kHz to 10 mHz, obtaining 10 points per decade. Frequency scans were carried out by applying ± 10 mV sinusoidal wave perturbation versus OCP.

## 3. Results and Discussion

### 3.1. Latex and Film Characterization

In this work, high biobased content latexes were produced using 2-OA and IBOMA as monomers. 2-OA is a soft monomer whereas IBOMA is a hard one; their homopolymers have a *T*_g_ of −44 °C and 150 °C, respectively. For coating formulations, the *T*_g_ of the used polymer should be below the application temperature (normally room temperature) in order to form a coherent film, but at the same time it should be high enough to produce good mechanical properties and avoid problems such as dirt pick up and blocking. The *T*_g_ of the polymers used for coatings is usually around 10–15 °C. Therefore, the 2-OA/IBOMA ratio used in this work was 58.5/41.5 wt% in order to obtain a copolymer with a *T*_g_ of 10 °C. Badia et al. [20] synthesized partially biodegradable waterborne coatings with a biocontent ranging from 30 to 65% using Ecomer^®^, an allyl polyglucoside maleic acid ester functional monomer, in combination with 2-OA, IBOMA and butyl acrylate (because Ecomer^®^ is commercialized as a solution in butyl acrylate). To the best of our knowledge, this is the first time that 2-OA and IBOMA are used as sole monomers in a coating formulation.

Table 2 shows the characterization of the synthesized latexes. There was no significant difference between the average size of the initial miniemulsion droplet and the final latex polymer particle, indicating that there was no secondary nucleation nor coagulation during the polymerization. Total conversion was achieved in both cases. Whereas no important coagulation was observed in the sample with no CeO_2_, less than 5% coagulum was obtained in the sample with 1 wbm % of CeO_2_, which can be attributed to a certain incompatibility between the CeO_2_ nanoparticles and the Sipomer PAM200, as observed previously with ZnO by Chimenti et al. [43].

**Figure 1:**
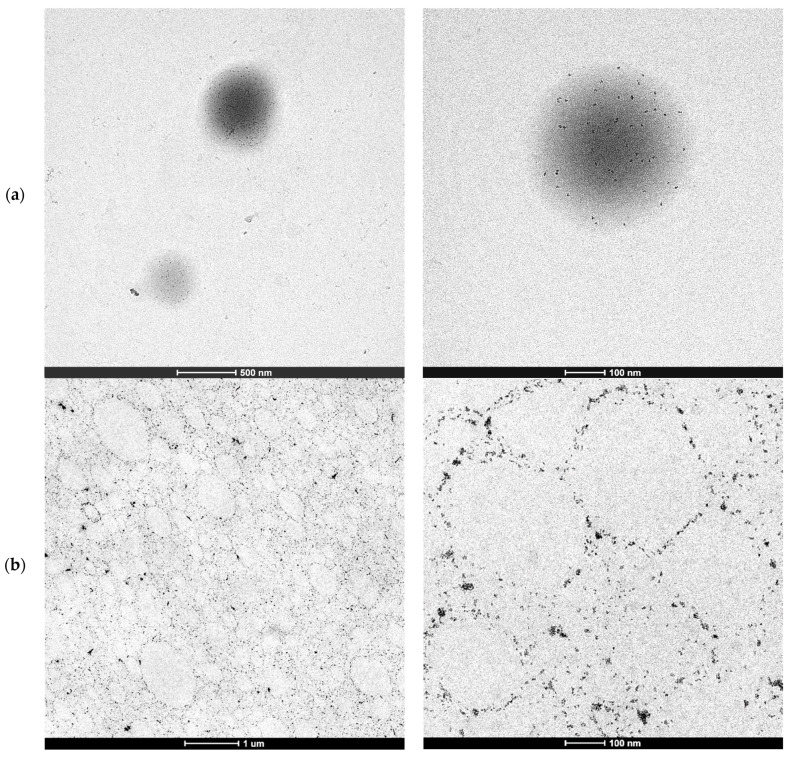
TEM micrographs of the hybrid CeO_2_-Bioacrylic latex polymer particles (**a**) and hybrid CeO_2_-Bioacrylic film (**b**).

Figure 1 shows the TEM micrographs of the water dispersed polymer particles containing 1 wbm % of CeO_2_ nanoparticles (a) and the cryosections of the film produced by casting such latex (b).

**Figure 2:**
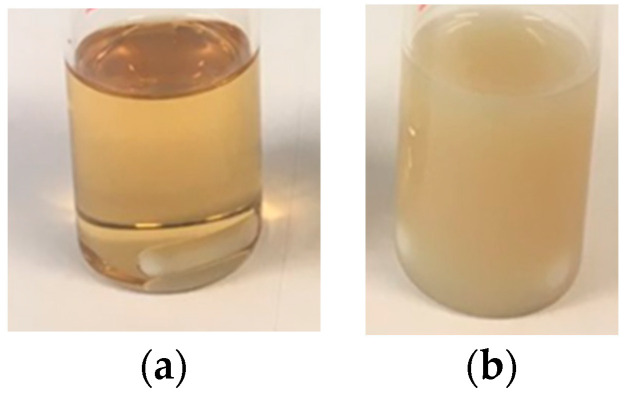
1 wbm% of CeO_2_ nanoparticles dispersed in 2-OA/IBOMA (**a**) and in 2-OA/IBOMA/AA (**b**).

Figure 1a,b show that the individual CeO_2_ nanoparticles did not aggregate during the polymerization but migrated to the surface of the polymer particles as in Pickering stabilized latexes. The lack of aggregation of the individual CeO_2_ nanoparticles was also proved by XRD of the hybrid CeO_2_-Bioacrylic films, by which an average CeO_2_ nanoparticle size of 6.8 nm, close to the original CeO_2_ size [44], was obtained by the use of the Scherrer equation (see Figure S1 in Supplementary Material). This is surprising because the CeO_2_ nanoparticles do disperse well in the monomer mixture (see Figure 2a with the transparent dispersion of CeO_2_ in 2-OA/IBOMA) as they do in a mixture of MMA/BA/AA [44]. Note that if acrylic acid was used with 2-OA and IBOMA, the CeO_2_ nanoparticles agglomerated (Figure 2b).

The morphology of the hybrid polymer particles and the film shown in Figure 1 were not expected because for a similar formulation with oil-based monomers (e.g., MMA/BA/AA), and the same CeO_2_ nanoparticles (although with the use of a conventional anionic surfactant, Dowfax 2A1), a single nanoparticle aggregate per polymer particle was found at the edge of the polymer particles [45,46,47,48]. A detailed monitoring of the evolution of the morphology by cryo-TEM demonstrated that the CeO_2_ nanoparticles were initially well dispersed inside the MMA/BA/AA droplets, but as polymerization proceeded, they aggregated due to the incompatibility between the formed copolymer and the modified CeO_2_ nanoparticle surface, leading to the formation of a single larger CeO_2_ nanoparticle aggregate [48].

**Figure 3:**
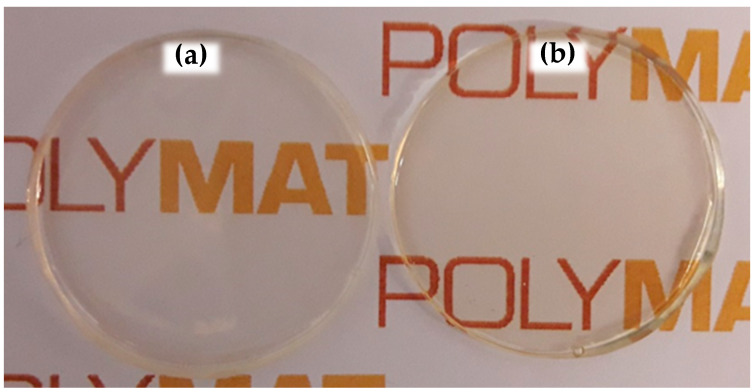
Free films formed at 24 ± 2 °C and 50 ± 5% relative humidity from (**a**) Bioacrylic latex and (**b**) hybrid CeO_2_-Bioacrylic latex.

Therefore, the interaction of the phosphate groups of the Sipomer PAM200 and the surface of the organically modified CeO_2_ nanoparticles (which was not disclosed by ALTANA), made the CeO_2_ nanoparticles to migrate to the monomer droplet/aqueous phase interface (polymer particle/aqueous phase after polymerization). This surfactant–nanoparticle interaction could be the reason for the decreased stabilization capability of the surfactant, causing the formation of some coagulum in this polymerization. Anyway, good quality clear films were obtained after casting Bioacrylic and CeO_2_-Bioacrylic latexes in silicone molds, even if the hybrid one had a slight yellowish color due to the presence of CeO_2_ nanoparticles (Figure 3).

**Figure 4:**
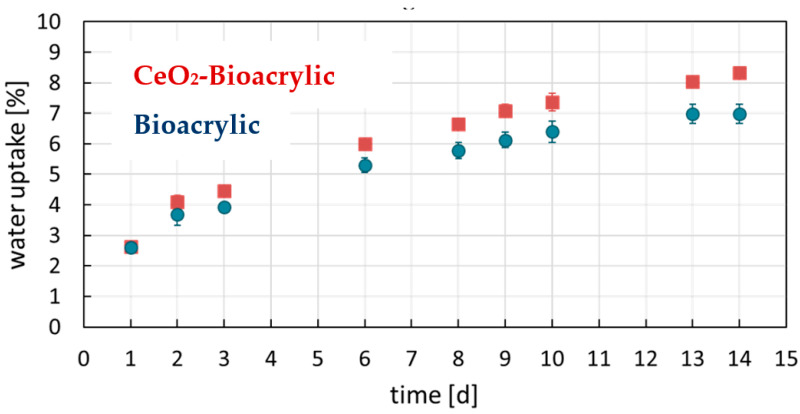
Water uptake experiment results of neat polymer film and the hybrid one.

Regarding the water sensitivity of the films, Figure 4 shows the results of the water uptake experiments. The final values as well as the water contact angle measurements are shown in Table 3.

Hydrophobic films were obtained, providing low water uptake values and high contact angle compared to values reported in the literature for acrylic latexes stabilized by polymerizable surfactant (18% of water uptake in 14 days and 75° contact angle for an MMA/BA acrylic binder [7,10]). The hydrophobicity of these films is due to the use of polymeric surfactants and the chemical nature of the copolymer. The addition of the CeO_2_ nanoparticles (1 wbm%) did not have a significant detrimental effect on the hydrophobic properties of the films.

### 3.2. Anticorrosion Properties

**Figure 5:**
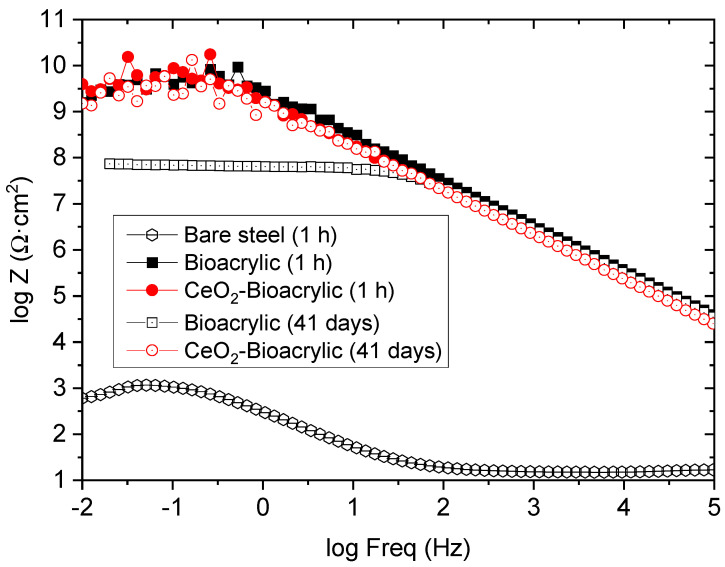
Bode plot of intact binders after 1 h and 41 days of exposure to 3.5 wt% NaCl solution.

In order to study the effect of the CeO_2_ nanoparticles, intact films (both neat and hybrid) with similar thickness (30–40 µm) were evaluated by EIS. Figure 5 shows the impedance diagrams after different exposure time (1 h and after 41 days, respectively). A capacitive behavior can be observed for both films at the beginning of the exposure (1 h), as an indication of the good barrier capabilities. Indeed, a single time constant is observed for both films, having an impedance modulus (|Z|) at low frequency (0.01 Hz) in the range 10^9^–10^10^ Ωcm^2^ (|Z|_0.01Hz_). It indicates an excellent barrier protection compared to acrylic waterborne coating without [49] and with a topcoat [50] or to epoxy coatings formulated with nano-CeO_2_ during exposure to NaCl electrolytes [41,51], where coatings were showing a much lower impedance value at shorter exposure time. However, this excellent barrier protection was only maintained after 41 days of exposure for the hybrid film. In fact, after that time, the Bioacrylic film decreased its impedance |Z|_0.01Hz_ in more than one order of magnitude, reaching a value around 10^8^ Ωcm^2^. The better performance of the hybrid coating can be justified by the physical blocking effect to the electrolyte diffusion thanks to the CeO_2_ nanoparticles [31,52], taking into account their stability in neutral to basic aqueous environemnts [53]. Therefore, the long-term durability shown by the hybrid film can be attributed to the inhibition effect of CeO_2_ nanoparticles located in the surface of the polymer particles.

**Figure 6:**
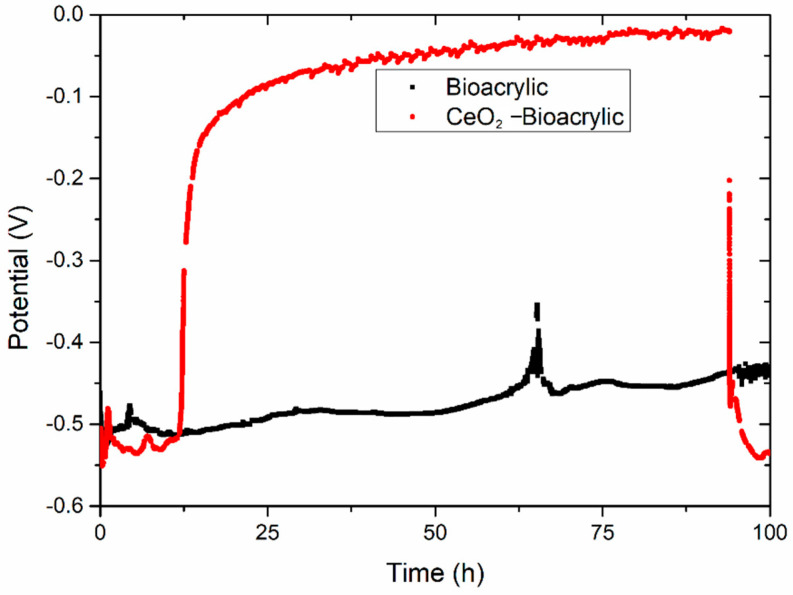
Open circuit potential of both Bioacrylic binders with an artificial defect after exposure to 3.5 wt% NaCl solution.

In order to confirm such hypothesis, an artificial defect has been created by laser on both films. The aim was to reach the metal/film interface in order to explore the inhibition capabilities of CeO_2_ nanoparticles [11]. Just immediately after provoking the defect, films were exposed to 3.5 wt% NaCl electrolyte. In addition to the EIS measurement per hour, OCP was monitored with time (Figure 6). Initially (after 10 h), a potential value around −0.50/−0.55 V was obtained for both systems. This potential value is typical of unprotected low carbon steel [36], and it confirms that the artificial defect was able to reach the interface. A monotonous increase of the potential was observed for the Bioacrylic film along the entire period of exposure (except for a random increase up to −0.36V at 65 h), reaching a value of −0.43 V after 100 h. In contrast, the CeO_2_-Bioacrylic film showed an exponential increase of the potential from −0.52/−0.51 V (11/12 h) up to −0.02 V (94 h), which is in agreement with the potential trend observed for a waterborne acrylic coating doped with CeO_2_ nanoparticles acting as corrosion inhibitor [54]. The quasi steady-state behavior (from 25 to 94 h) around −0.1/0.02V was also observed on steel protected with metallic coatings (nickel nanocomposite) having CeO_2_ nanoparticles grafted with ferrocene, where the formation of a passive layer provided less negative (i.e., anodic) OCP values over a large period of time (30 days) [55]. Finally, an abrupt drop of the potential suddenly occurred, reaching a similar potential value to the one obtained at beginning of the test (−0.55 V) after 100 h.

**Figure 7:**
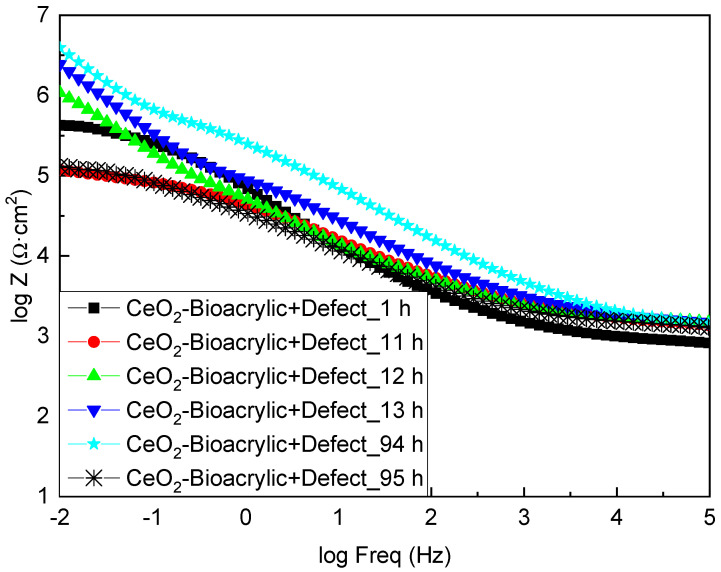
Bode plots of CeO_2_—Bioacrylic binder with an artificial defect after different immersion times in 3.5 wt% NaCl solution.

Apparently, the completely different behavior shown by both films, in terms of OCP, might be attributed to the role of CeO_2_ as a corrosion inhibitor. Therefore, a tailored analysis of the EIS diagrams was done for the different times of interest (i.e., before and after the variation of the potential values in Figure 6). Figure 7 shows the EIS diagrams for the CeO_2_-Bioacrylic film with an artificial defect after 1, 11, 12, 13, 94 and 95 h of exposure, respectively. If the impedance modulus is compared at low frequency (|Z|_0.01Hz_) for each time, it can be observed that |Z|_0.01Hz_ was 6 × 10^5^ Ωcm^2^ after 1 h of exposure. It was decreasing slightly to around 10^5^ Ωcm^2^ after 11 h, indicating that the corrosion process was taking place up to then. However, this trend was drastically changed when a sudden increase of |Z|_0.01Hz_ occurred at 12 h—impedance reaching 10^6^ Ωcm^2^ and 6 × 10^6^ Ωcm^2^ at 12 and 13 h, respectively. A steady state value was maintained above 5 × 10^6^ Ωcm^2^ from 13 h until 94 h of exposure, thanks to the corrosion inhibition capabilities of CeO_2_ nanoparticles. This is in agreement with the delay of the corrosion onset shown on coatings formulated with CeO_2_ nanoparticles having an artificial defect: the corrosion activity is limited to the vicinity of the defective area according to the results obtained by localized electrochemical techniques [56]. Finally, the impedance dropped down again to the minimum value (10^5^ Ωcm^2^), indicating that most probably the corrosion inhibition was exhausted and the metal/film interface on the artificial defect was not protected anymore.

**Figure 8:**
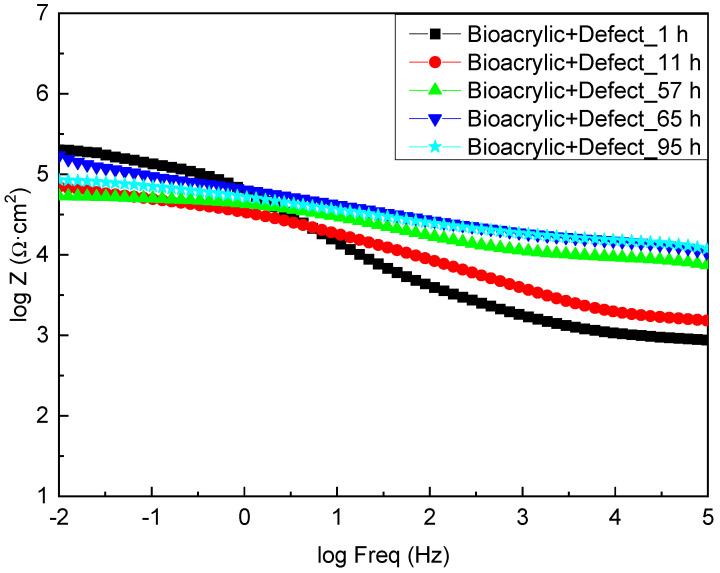
Bode plots of Bioacrylic binder with an artificial defect after different immersion time in 3.5 wt% NaCl solution.

On the other hand, Figure 8 shows the EIS diagrams for the Bioacrylic film with an artificial defect after 1, 11, 57, 65 and 95 h of exposure to salty water, respectively. Initially, a similar value of the impedance modulus was obtained (3 × 10^5^ Ωcm^2^) at low frequency (|Z|_0.01Hz_) compared to the hybrid film (6 × 10^5^ Ωcm^2^), which also decreased to values below 10^5^ Ωcm^2^ after 11 h of exposure. In contrast to the CeO_2_-Bioacrylic film, the impedance value slightly varied until a minimum was reached at 57 h (7 × 10^4^ Ωcm^2^) due to the absence of any corrosion inhibition into the film. The slight |Z|_0.01Hz_ increase (3 × 10^5^ Ωcm^2^) observed after 65 h may be related to the presence of corrosion products that are blocking the pinhole/damage that was created with the artificial defect. It can be observed that |Z|_0.01Hz_ remains in a very narrow range (7 × 10^4^/3 × 10^5^ Ωcm^2^) during the entire test, and it confirms that no protection can be achieved in the absence of CeO_2_ nanoparticles in the film formulation. Indeed, a |Z|_0.01Hz_ value of 9 × 10^4^ Ωcm^2^ was obtained after 95 h of exposure, indicating the absence of protection in the interface. Therefore, these results confirm that homogeneously distributed CeO_2_ nanoparticles are required into the film to provide an efficient corrosion protection of the metal/film interface.

## 4. Conclusions

Novel waterborne hybrid CeO_2_ biobased acrylic binders were synthesized by miniemulsion polymerization. CeO_2_ nanoparticles interacted with the phosphate moieties of the surfactant and migrated to the interphase leading to polymer particles with Pickering morphology. Thus, CeO_2_ nanoparticles did not aggregate and were well distributed in the surface of the polymer particles. The biobased acrylic copolymer, together with the phosphate surfmer used in the synthesis of the polymer particles, produced films with low water uptake and high contact angle to water. EIS results also showed enhanced barrier properties of both films, independently of the presence of CeO_2_ nanoparticles. However, the long-term durability of the intact hybrid film was higher than the neat one. This can be attributed to the corrosion inhibition capabilities of CeO_2_ nanoparticles, according to the results obtained when both types of films had an artificial defect. EIS diagrams showed an increase of the impedance modulus in one order of magnitude (|Z|_0.01Hz_ went from 6 × 10^5^ to 6 × 10^6^ Ωcm^2^ after 13 h) thanks to key role of CeO_2_ nanoparticles. In fact, the neat film did not show any protection of the interface in the presence of an artificial defect.

## Figures and Tables

**Table 1:** Formulation used for the miniemulsion polymerization. The target solids content was 40 wt%.

	Component	wt%
Organic phase	IBOMA	16.6
	2-OA	23.4
	Octadecyl acrylate *	4
	CeO_2_ *	0–1
	AIBN *	1
Water phase	Water	60
	Sipomer^®^ PAM200 *	2

* Weight % with respect to the total weight of monomers (wbm %).

**Table 2:** Characterization of synthesized latexes. Both latexes have a pH value of 7.

Latex	CeO_2_ (wbm %)	Droplet Diameter (nm)	Particle Diameter (nm)
Bioacrylic	-	204 ± 5	200 ± 2
CeO_2_-Bioacrylic	1	218 ± 5	230 ± 2

**Table 3:** Water sensitivity of the neat polymer film and the hybrid one.

Film	Contact Angle to Water (°)	Water Uptake after 14 Days (%)
Bioacrylic	92 ± 3	7.0 ± 0.3
CeO_2_-Bioacrylic	92 ± 1	8.3 ± 0.2

## Supplementary Materials

The following are available online at https://www.mdpi.com/2073-4360/13/6/848/s1, Figure S1: XRD patterns of neat Byoacrylic and hybrid CeO_2_-Bioacrylic film.
