# Supplementary material for: Assessing the Effect of CeO2 Nanoparticles as Corrosion Inhibitor in Hybrid Biobased Waterborne Acrylic Direct to Metal Coating Binders
Source: Polymers (Basel). 2021 Mar 10;13(6):848. doi: 10.3390/polym13060848 (PMC7999048; doi:10.3390/polym13060848)
Supplement: Supplementary file 1 [file polymers-13-00848-s001.pdf]

polymers

MDPI

Supplementary Material

Assessing the Effect of CeO_2 Nanoparticles as Corrosion Inhibitor in Hybrid Biobased Waterborne Acrylic Direct to Metal Coating Binders

Edurne González [1], Robin Stuhr [1], Jesús Manuel Vega [2], Eva García-Lecina [2], Hans-Jürgen Grande [2,3], Jose Ramon Leiza [1] and María Paulis [1,*]

[1] POLYMAT, Applied Chemistry Department, Faculty of Chemistry, University of the Basque Country (UPV/EHU), 20018 Donostia-San Sebastián, Spain; edurne.gonzalezg@ehu.eus (E.G.); robin.stuhr@studium.uni-hamburg.de (R.S.); jrleiza@ehu.eus (J.R.L.)
[2] CIDETEC, Basque Research and Technology Alliance (BRTA), Paseo Miramón 196, 20014 Donostia-San Sebastián, Spain; jvega@cidetec.es (J.M.V.); egarcia@cidetec.es (E.G.-L.); hgrande@cidetec.es (H.-J.G.)
[3] POLYMAT, Polymers and Advanced Materials: Physics, Chemistry and Technology Department, Faculty of Chemistry, University of the Basque Country (UPV/EHU), 20018 Donostia-San Sebastián, Spain
* Correspondence: maria.paulis@ehu.eus

Figure S1 presents the XRD diffractograms of the neat and hybrid Bioacrylic films prepared in this study.

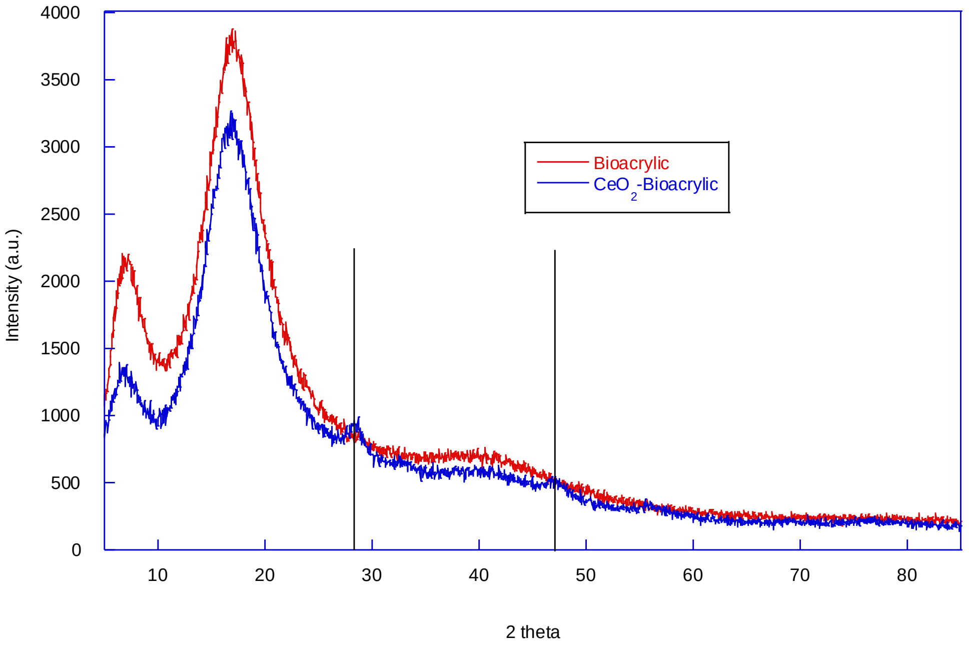

Figure S1. XRD of the neat Bioacrylic film and the hybrid CeO_2-Bioacrylic film.

The two main peaks of cubic crystalline form of CeO_2 can be seen in the diffractogram of the hybrid film (at 28° (111) and at 47° (220)). Using Scherrer equation: $D = \frac{0.98.\lambda}{B.cos\theta}$ to obtain the mean diameter of the CeO_2 nanoparticles (D), being λ the wavelength of the CuK radiation (0.15406 nm) and B the line broadening at half height in radians, we retrieve a mean D (diameter) of 6.8 nm from the broadening of both peaks. So, the initial CeO_2 nanoparticles (8 nm in average as measured in the initial dispersion in the organic solvents) are not agglomerating in this system.
